# Effects of Intravenous Infusion With Sodium Butyrate on Colonic Microbiota, Intestinal Development- and Mucosal Immune-Related Gene Expression in Normal Growing Pigs

**DOI:** 10.3389/fmicb.2018.01652

**Published:** 2018-07-20

**Authors:** Xue Chen, Jumei Xu, Yong Su, Weiyun Zhu

**Affiliations:** Jiangsu Key Laboratory of Gastrointestinal Nutrition and Animal Health, College of Animal Science and Technology, Nanjing Agricultural University, Nanjing, China

**Keywords:** colonic microbiota, growing pigs, gut development, intravenous butyrate, mucosal immune

## Abstract

This study aimed to investigate effects of intravenous infusion with sodium butyrate (SB) on colonic microbiota, intestinal mucosal immune and intestinal development in normal growing pigs. Twelve crossbred barrows (Duroc × Landrace × Large White) fitted with a medical polyethylene cannula via internal jugular vein were daily infused with 10 ml SB (200 mmol/l) or the same volume of physiological saline for 7 days. Results showed that SB infusion had no effects on the short-chain fatty acids concentrations and the number of total bacteria, but significantly increased the microbial richness estimators (ACE and Chao1), and the abundance of genera related to Clostridiales order in the colonic digesta (*P* < 0.05). SB infusion significantly up-regulated the mRNA expression of monocarboxylate transporter 1 (*MCT1*) in the colon, while no change was found in the ileum. Only the relative mRNA of pro-inflammatory cytokine *IL-6* gene was decreased significantly in the ileum by SB infusion. On the contrary, in the colon, SB infusion significantly decreased the gene expression of histone deacetylase 1 (*HDAC1*) and pro-inflammatory cytokines *IL-6, IL-18, IL-12p40*, and *TNF-α* (*P* < 0.05), but significantly increased the secretory immunoglobulin A (sIgA) concentration, the gene expression of anti-inflammatory cytokine *IL-10*, and the expression of intestinal development-related gene zonula occludens-1 (*ZO-1*), occludin, and epidermal growth factor (*EGF*) (*P* < 0.05). The results suggest that systemic SB can modify colonic microbial composition, regulate the inflammatory cytokine- and intestinal development-related gene expression in pigs under the normal physiological condition. This study may provide an alternative strategy for improving the intestinal health of normal piglets.

## Introduction

Microbes in the intestine degrade a variety of plant polysaccharides and other dietary substances (Ley et al., 2008). This not only enhances host digestive efficiency, but also provides energy source for the microbes (Duncan et al., 2007). Researchers regard the gut microbiota as a forgotten organ in the host due to its capacity to communicate with one another and the host by different ways and thus affect the host metabolic, nutritional, physiological, and immunological processes (Round and Mazmanian, 2009; Cerf-Bensussan and Gaboriau-Routhiau, 2010). Therefore, maintaining the balance of the microbial ecosystem is the key to keep host health (Biagi et al., 2012).

Butyrate, a short-chain fatty acid (SCFA), is mainly produced by microbial fermentation of diet fiber in the large intestine of monogastric animals (Von Engelhardt et al., 1989). Butyrate has shown to be related with several beneficial effects on gut health because it can be quickly absorbed by the epithelial cells of the terminal ileum and large intestine, especially in the colon, and thus provides energy for the epithelial cells to stimulate the epithelial cells proliferation, differentiation, and maturation, and reduce cell apoptosis (Cummings and Macfarlane, 1997). Previous study showed that sodium butyrate (SB) supplementation in the diet had benefit effect on villous-crypt architecture and thus improved gut barrier function and the host digestive efficiency (Galfi and Bokori, 1990; Wang et al., 2012). The anti-bacteria and anti-inflammatory properties was another reason why SB was used extensively by the livestock industry (Chang et al., 2014). It has been demonstrated that oral administration of SB for neonatal pigs significantly improves the diversity of microbiota in the stomach and colon, and decreases the gene expression of pro-inflammatory cytokines in the ileum (Xu et al., 2016). At the extraintestinal level, parenteral butyrate or SCFAs were reported to be beneficial to the gastrointestinal structure (Koruda et al., 1990; Rolandelli et al., 1997; Tappenden et al., 1998; Bartholome et al., 2004), function (Tappenden et al., 1997; Tappenden and Mcburney, 1998; Drozdowski et al., 2002), and mucosal immunity (Milo et al., 2002; Murakoshi et al., 2011) in mouse, rat, or pig models. However, little information is available on the impact of parenteral butyrate on gut microbiota and their subsequent role in host gut health.

While the beneficial roles of SB combined with parenteral nutrition in gut health were observed in most studies with animal models of intestine injury (Tappenden et al., 1997; Milo et al., 2002; Bartholome et al., 2004; Murakoshi et al., 2011), it is not clear whether the intravenous infusion with SB can play the similar roles in normal animals. In this study, we hypothesize that additional intravenous butyrate can affect the gut function and mucosal immunity, then indirectly modify the microbial community, and eventually impact the gut health of the normal piglets. Therefore, the aim of this study was to investigate the effects of additional intravenous infusion with SB on gut microbiota, mucosal immune and intestinal development in growing pigs in a short-term experiment, which may be a reference in the future study on developing alternative strategies for improving the intestinal health of normal piglets.

## Materials and Methods

### Ethics Statement

The experiment was approved and conducted under the supervision of the Animal Care and Use Committee of Nanjing Agricultural University (Nanjing, Jiangsu province, China). All pigs were raised and maintained on a local commercial farm under the care of the Animal Care and Use Guidelines of Nanjing Agricultural University.

### Animals, Housing, and Experimental Design

Twelve crossbred growing barrows (Duroc × Landrace × Large White; initial body weight = 19.53 ± 1.35 kg) from the research pig farm (Nanjing Agricultural University) were used in the present study. Pigs were surgically fitted with a medical polyethylene cannula (inside diameter 2 mm and outside diameter 3 mm) via internal jugular vein after anesthesia to allow for the infusion of SB. One week prior to surgery, pigs were moved into 1.0 × 1.2 m individual pens for adaptation to the new environment, where they were housed for the duration of the experiment. During the 1-week recovery period after surgery, pigs consumed a commercial grower diet (Supplementary Table 1) (metabolizable energy = 3.19 Mcal/kg; crude protein = 16.8%, as-fed basis). Feed amounts were gradually increased after surgery until reaching pre-surgery levels. Following recovery, pigs (body weight = 23.70 ± 1.29 kg) were randomly allocated into the SB and control (CO) groups. Each group consisted of six replicates (pens), with one pig per pen. Pigs in the SB group were infused with 10 ml SB (200 mmol/l, PH 7.4, dissolved in sterile water) via internal jugular vein at 9:00 am each day, while the CO group was treated with the same volume of physiological saline (0.9% NaCl, pH 7.4). The dosage was chosen according to previous studies in pig including butyrate through parenteral nutrition (Bartholome et al., 2004) or through oral administration (Xu et al., 2016). The intravenous treatment lasted for 7 days. Pigs had unlimited access to feed and water throughout the experiment.

### Sampling

On day 8, all the pigs were euthanized with a jugular vein injection of 4% sodium pentobarbital solution (40 mg/kg body weight). Pigs were weighed before euthanasia to determine average daily weight gain. The digesta in the distal ileum and proximal colon were collected, immediately snapped frozen in liquid nitrogen, and stored at -28°C. Analysis of microbial composition and SCFAs were performed on samples in the colon, and secretory immunoglobulin A (sIgA) concentration was determined on samples both in the ileum and colon. To determine the expression of inflammatory cytokine-, tight junction protein-, and intestine development-related genes in the ileum and colon, the luminal fluid was drained, distal segments of distal ileum and proximal colon (3–4 cm) were excised and washed with sterile phosphate buffer solution (PBS, pH 7.0), then the tissues were immediately snapped frozen in liquid nitrogen, and stored at -80°C.

### Short-Chain Fatty Acid Concentration Analysis

The SCFA concentrations in the proximal colon were determined by using a capillary column gas chromatograph (GC-14B, Shimadzu, Japan; Capillary Column: 30 m × 0.32 mm × 0.25 μm film thickness) according to the description in a previous study (Zhou et al., 2016).

### Illumina MiSeq Sequencing 16S rDNA Gene in the Colon and Bioinformatics Analysis

The bacterial DNA was isolated from proximal colonic digesta using a commercially available stool DNA extraction kit according to the manufacturer’s instructions (QIAamp DNA Stool Mini Kit, Qiagen, Hilden, Germany). The concentration of extracted DNA was determined by using a NanoDrop 1000 spectrophotometer (Thermo Fisher Scientific Inc., Wilmington, DE, United States). The V4–V5 region of the bacterial 16S rRNA gene was amplified by polymerase chain reaction (PCR) using bacterial universal primers 515F (5′-GTGCCAGCMGCCGCGG-3′) and 907R (5′-CCGTCAATTCMTTTRAGTTT-3′) according to the description of previous study (Xiong et al., 2012). Purified amplicons were pooled in equimolar and paired-end sequenced (2 × 250) on an Illumina MiSeq platform according to the standard protocols at the Majorbio Bio-Pharm Technology (Shanghai, China).

Raw fastq files were demultiplexed and quality-filtered using QIIME (version 1.17) with the following criteria: the 250 bp reads were truncated at any site receiving an average quality score <20 over a 10 bp sliding window, discarding the truncated reads that were shorter than 50 bp; exact barcode matching, two nucleotide mismatch in primer matching, reads containing ambiguous characters were removed; and only sequences that overlap longer than 10 bp were assembled according to their overlap sequence. Operational taxonomic units (OTUs) were clustered with 97% similarity cutoff using UPARSE (version 7.1)^1^, and chimeric sequences were identified and removed using UCHIME. To assess bacterial diversity among samples in a comparable manner, a randomly selected, 31887-sequence (the lowest number of sequences in the 12 samples) subset from each sample was compared for the phylogenetic affiliation by RDP Classifier^2^ against the Silva (SSU115) 16S rRNA database using a confidence threshold of 70% (Amato et al., 2013). We also calculated the sequencing depth index (coverage percentage) using Good’s method (Good, 1953), the abundance-based coverage estimator (ACE), the bias-corrected Chao1 richness estimator, the Pielou evenness index (E), and the Shannon and Simpson diversity indices using the MOTHUR program^3^ (Schloss et al., 2009). The raw sequencing reads were submitted to Sequencing Read Archive (SRA) database under the accession id: SRP151524. The Bray–Curtis similarity clustering analysis of the abundance of OTUs was used to perform a principal coordinates analysis (PCoA) (Bray and Curtis, 1957). The relative abundance at the phylum and genus levels was compared between the two groups, the top 30 most abundant genera were defined as predominant genera, and sorted for the comparison.

### Quantification of Total Bacteria by Real-Time PCR

Total bacteria primer set Bact1369 (5′-CGGTGAATACGTTCYCGG-3′) and Prok1492 (5′-GGWTACCTTGTTACGACTT-3′) was used for the quantification of total bacteria in the colon of pigs (Suzuki et al., 2000). Real-time PCR was performed on an Applied Biosystems 7300 real-time PCR system (Applied Biosystems, United States) using SYBR Green as the fluorescent dye as described by Sun et al. (2015). The PCR amplification was performed with an initial denaturation step of 95°C for 3 min, followed by 40 cycles of 95°C for 15 s, 56°C for 30 s and 72°C for 30 s. Standard curves were generated with 10-fold serial dilutions of 16S rRNA genes amplified from a *Lactobacillus* strain. The number of total bacterial 16S rRNA gene copy was plotted against the CT value, and expressed as Log_10_ 16S rRNA gene copies per gram of fresh sample.

### Analysis of sIgA Concentration

The digesta (0.5 g) in the distal ileum and proximal colon were mixed with 4.5 ml physiological saline (0.9% NaCl, pH 7.4) through tissue homogenate. Then, sIgA concentration was determined by ELISA kit (Jiancheng Biochemical Reagent Company, Nanjing, China).

### RNA Extraction, cDNA Synthesis, and Real-Time RT-PCR

Total RNA was extracted from distal segments of distal ileum and proximal colon using TRIzol reagent (Invitrogen, China), and quantified using a NanoDrop 1000 spectrophotometer (Thermo Fisher Scientific Inc., Wilmington, DE, United States). The absorption ratio (260:280 nm) of all the samples was between 1.8 and 2.0. One microgram RNA was reverse-transcribed with standard reagents (Biocolors, China). The primers for SCFA uptake [monocarboxylate transporter 1 (*MCT1*)] (Haenen et al., 2013), pro-inflammatory cytokines (*IL-6, IL-8, IL-1β, IL-12p40, IL-18, TNF-α*, and *IFN-γ*) (Pié et al., 2004; Feng et al., 2014; Tudela et al., 2015), anti-inflammatory cytokine (*IL-10* and *TGF-β*) (Pieper et al., 2012; Feng et al., 2014), gene expression regulator [histone deacetylase 1 (*HDAC1*)] (Lee et al., 2011), tight junction protein genes [zonula occludens-1 (*ZO-1*) and occludin] (Feng et al., 2014), intestine development-related genes [insulin-like growth factor-1 (*IGF-1*), preproglucagon, *IGF-1R*, and epidermal growth factor (*EGF*) (Xiang, 2011; Han et al., 2012) and housekeeping genes (β-actin and *GAPDH*) (Li et al., 2016; Zhang et al., 2017) used in this study are presented in Supplementary Table 2. The target genes and housekeeping genes were measured by quantitative real-time PCR with SYBR Green (Roche, Switzerland) and fluorescence was detected on an ABI 7300 sequence detector. The reaction system included 10 μl SYBR, 2 μl DNA (100 ng/μl), 0.4 μl Rox dye, 0.4 μl forward and reserve primers (10 mmol/μl), and 6.8 μl double distilled water. Samples were incubated in the ABI 7300 sequence detector for an initial denaturation at 95°C for 10 min, followed by 35 PCR cycles of 95°C for 15 s, 60°C for 1 min, and 72°C for 1 min. Of the three candidate housekeeping genes, β-actin was finally used for the accurate normalization by NormFinder software as described by Andersen et al. (2004). The fold change was calculated using the 2^-ΔΔCt^ method, presented as the fold-expression change in SB group relative to CO group after normalization to the endogenous control, β-actin. Amplification of specific transcripts was confirmed by melting curve profiles at the end of each PCR.

### Statistical Analysis

Power calculations before the start of the study had identified a required sample size of six piglets per treatment group in order to enable detection of an effect size of 2.32 SD for microbial and gene expression data with 95% power and a type I error of 5% by using G^∗^Power Data Analysis (Faul et al., 2007). Data were analyzed by SPSS 17.0 as a completely randomized design, considering the SB treatment as the main effect. The microbial data were analyzed by using the non-parametric Mann–Whitney *U*-test for independent samples. The data of average daily weight gain, SCFAs and sIgA concentrations, inflammatory cytokine-, tight junction-, and intestine development-related genes were evaluated by Student’s *t*-test. Data were presented as group mean ± SE, significant differences were declared when *P* < 0.05 and a trend when *P* < 0.10. The correlations between the colonic microbial composition (relative abundance of genus higher than 0.1%) and inflammatory cytokines-, tight junction protein-, and intestine development-related gene expression which were significantly affected by SB treatment were assessed by Pearson’s correlation test using GraphPad Prism version 5.00 (GraphPad Software, San Diego, CA, United States).

## Results

### Average Daily Weight Gain of Pigs and SCFA Concentrations

No difference was found in the average daily weight gain of pigs between the SB and CO groups (0.82 ± 0.08 kg vs 0.81 ± 0.06 kg). Intravenous infusion with SB did not affect the SCFA concentrations in the colon of pigs (**Table 1**).

**Table 1:** Short-chain fatty acid (SCFA) concentrations (μmol/g) in the colon of pigs in the sodium butyrate (SB) and control (CO) groups.

Items	CO	SB	*P*-value
Total SCFA	69.63 ± 4.14	62.77 ± 4.68	0.305
Acetate	41.25 ± 2.22	37.34 ± 2.11	0.238
Propionate	17.88 ± 1.84	16.00 ± 2.76	0.586
Butyrate	7.20 ± 0.80	6.02 ± 0.56	0.259
Isovalerate	1.23 ± 0.09	1.37 ± 0.11	0.317
Valerate	1.19 ± 0.07	1.00 ± 0.13	0.233
Isobutyrate	0.88 ± 0.20	1.04 ± 0.11	0.501

*Values are expressed as mean ± SE, *n* = 6.*

### Microbial Composition

**FIGURE 1:**
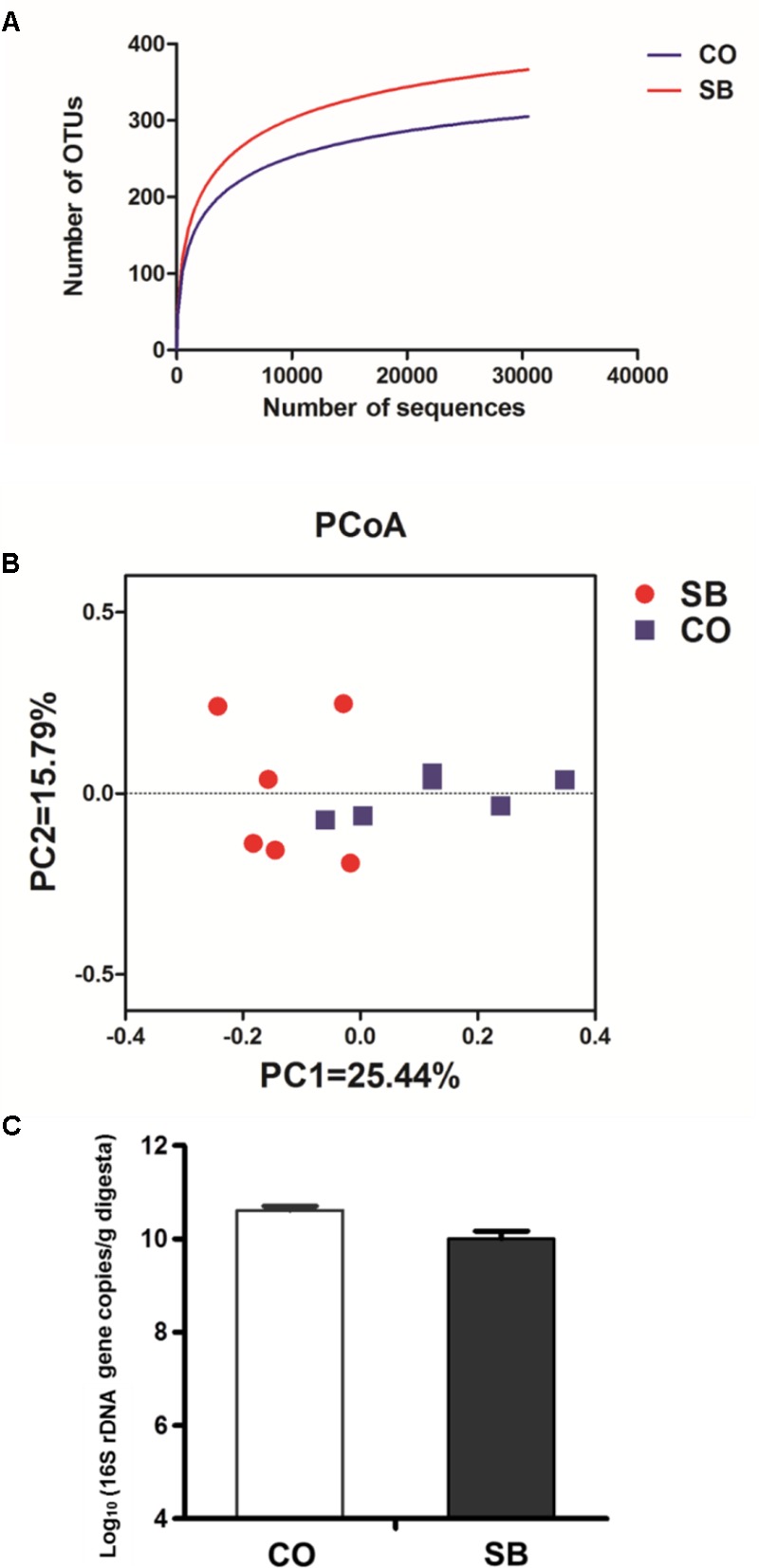
Effects of intravenous infusion with sodium butyrate (SB) on colonic microbiota of pigs. **(A)** Rarefaction curves (mean curves for the six samples/group) plotting the number of phylotypes found in the 16S rDNA gene libraries by the number of sequences from microbiota in the colonic digesta of pigs in the SB and control (CO) groups. **(B)** Principal coordinate analysis (PCoA) of colonic bacterial communities in the SB and CO groups. **(C)** The number of 16S rDNA gene copies of total bacteria in the colon of pigs in the SB and CO groups. Values are expressed as mean ± SE, *n* = 6.

Across all 12 samples, 446,252 quality sequences were classified as being bacteria with a read length higher than 250 bp. The average length of the quality sequences was 417 bp. The rarefaction curves (mean curves for the six samples/group) generated by MOTHUR plotting the number of OTUs by the number of reads tended to approach the saturation plateau (**Figure 1A**). The statistical estimates of species richness and diversity indices for 31887-sequence subsets from each sample at a genetic distance of 3% are presented in **Table 2**. SB infusion significantly increased the richness estimators (ACE and Chao1) in the digesta samples (*P* < 0.05), but had no effect on the coverage percentage, evenness index (E), and diversity indices (Shannon and Simpson) (*P* > 0.10). PCoA of colonic bacterial communities showed that samples in the CO group gathered together although the microbial clusters of the two groups were not completely separated (**Figure 1B**).

**Table 2:** Richness estimators, diversity and evenness indices of the 16S rRNA gene libraries from microbiota in the colon of pigs in the sodium butyrate (SB) and control (CO) groups.

Items		CO	SB	*P*-value
Richness estimators	Chao1	170.33 ± 9.91	208.05 ± 4.76	0.015
	Ace	151.63 ± 12.73	179.28 ± 5.97	0.015
Diversity index	Shannon	3.92 ± 0.10	4.09 ± 0.04	0.240
	Simpson	0.03 ± 0.01	0.03 ± 0.00	0.589
Evenness index	Pielou E	0.71 ± 0.03	0.71 ± 0.04	0.881
Coverage (%)		99.87 ± 0.03	99.87 ± 0.01	0.672

*Values are expressed as mean ± SE, *n* = 6.*

At the phylum level, Firmicutes and Bacteroidetes were the most predominant phyla in the colon (**Table 3**). Infusion with SB tended to decrease the relative abundance of Bacteroidetes (*P* = 0.065) and to increase the proportion of Firmicutes (*P* = 0.093) and Spirochaetae (*P* = 0.093). Genus-level analysis of the top 30 most abundant genera revealed that unclassified Clostridiales, unclassified Ruminococcaceae, *Clostridium sensu stricto*, and *Anaerotruncus* in the colon were significantly increased in relative abundance by the SB infusion, whereas the abundance of *Alloprevotella, Subdoligranulum*, and *Blautia* was decreased (*P* < 0.05) (**Table 4**).

**Table 3:** Relative abundance of microbial phylum (percentage) in the colon of pigs in the sodium butyrate (SB) and control (CO) groups.

Items	CO	SB	*P*-value
Bacteroidetes	50.97 ± 3.29	40.29 ± 3.49	0.065
Firmicutes	47.84 ± 3.27	57.34 ± 2.6	0.093
Tenericutes	0.49 ± 0.33	0.54 ± 0.07	0.132
Proteobacteria	0.38 ± 0.13	1.30 ± 1.00	0.818
Actinobacteria	0.14 ± 0.02	0.22 ± 0.07	0.310
Unclassified	0.09 ± 0.06	0.20 ± 0.08	0.485
Cyanobacteria	0.07 ± 0.03	0.07 ± 0.03	0.818
Spirochaetae	0.00 ± 0.00	0.03 ± 0.02	0.093

*Values are expressed as mean ± SE, *n* = 6.*

**Table 4:** Relative abundance (percentage) for the top 30 most abundant genera in the colon of pigs in the sodium butyrate (SB) and control (CO) groups.

Bacterial taxa	CO	SB	*P*-value
Unclassified Prevotellaceae	12.87 ± 0.82	11.49 ± 1.84	1.000
*Prevotella*	10.94 ± 2.88	8.13 ± 1.61	0.589
*Lactobacillus*	10.35 ± 3.48	4.34 ± 2.04	0.310
*Alloprevotella*	9.71 ± 0.92	4.2 ± 0.66	0.002
Unclassified Bacteroidales	9.35 ± 0.65	8.82 ± 1.15	0.589
Unclassified Clostridiales	6.96 ± 0.78	9.85 ± 1.34	0.041
Unclassified Ruminococcaceae	5.76 ± 0.88	9.24 ± 1.09	0.041
*Bacteroides*	5.00 ± 1.23	2.35 ± 0.26	0.065
*Clostridium sensu stricto*	3.33 ± 0.94	6.20 ± 0.82	0.041
Unclassified Lachnospiraceae	2.82 ± 0.82	5.35 ± 0.90	0.093
*Streptococcus*	2.79 ± 0.76	6.89 ± 2.32	0.240
*Coprococcus*	2.40 ± 0.41	1.81 ± 0.61	0.180
*Terrisporobacter*	2.32 ± 0.74	2.63 ± 0.27	0.485
Unclassified Rikenellaceae	2.00 ± 0.64	3.01 ± 0.51	0.180
*Phascolarctobacterium*	1.33 ± 0.13	0.88 ± 0.20	0.093
*Subdoligranulum*	1.33 ± 0.30	0.67 ± 0.27	0.026
[Eubacterium] coprostanoligenes group	1.23 ± 0.44	1.41 ± 0.28	0.818
*Parabacteroides*	1.00 ± 0.24	1.93 ± 0.43	0.093
*Blautia*	1.00 ± 0.12	0.58 ± 0.10	0.041
*Faecalibacterium*	0.98 ± 0.13	0.78 ± 0.17	0.589
*Ruminiclostridium*	0.95 ± 0.14	1.22 ± 0.23	0.485
*Lachnoclostridium*	0.62 ± 0.15	0.49 ± 0.06	0.394
Unclassified Mollicutes	0.45 ± 0.32	0.47 ± 0.08	0.132
*Ruminococcus*	0.43 ± 0.14	0.69 ± 0.15	0.240
*Oscillospira*	0.41 ± 0.11	0.48 ± 0.10	0.485
*Anaerotruncus*	0.29 ± 0.04	0.65 ± 0.12	0.015
*Oscillibacter*	0.28 ± 0.05	0.49 ± 0.08	0.132
*Anaerovibrio*	0.28 ± 0.15	0.41 ± 0.30	0.818
Unclassified Erysipelotrichales	0.18 ± 0.06	0.35 ± 0.06	0.132
*Leeia*	0.01 ± 0.01	0.99 ± 0.99	0.937

*Values are expressed as mean ± SE, *n* = 6.*

Because MiSeq sequencing analysis can only reflect the relative abundance of bacteria, quantitative real-time PCR was used to determine the number of 16S rRNA gene copies of bacteria in the colon of pigs. As shown in **Figure 1C**, SB infusion had no effect on the total numbers of bacteria in the colon of pigs (*P* > 0.10).

### sIgA Concentrations

**FIGURE 2:**
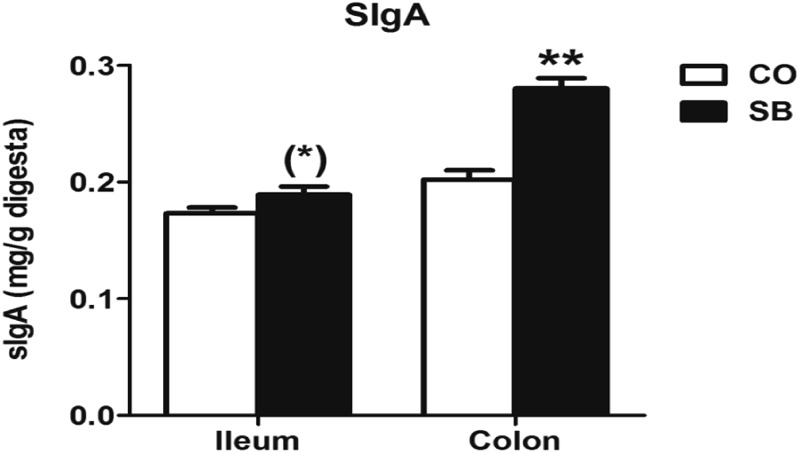
The concentration of secretory immunoglobulin A (sIgA) in the ileal and colonic digesta of pigs in the sodium butyrate (SB) and control (CO) groups. Values are expressed as mean ± SE, *n* = 6. ^∗∗^*P* < 0.01, (^∗^)0.05 ≤*P* < 0.1.

As shown in **Figure 2**, SB infusion significantly increased the sIgA concentration in the colonic digesta (*P* < 0.05), and tended to increase sIgA concentration in the ileum (*P* = 0.089).

### Gene Expression of *MCT1* and *HDAC1*

**FIGURE 3:**
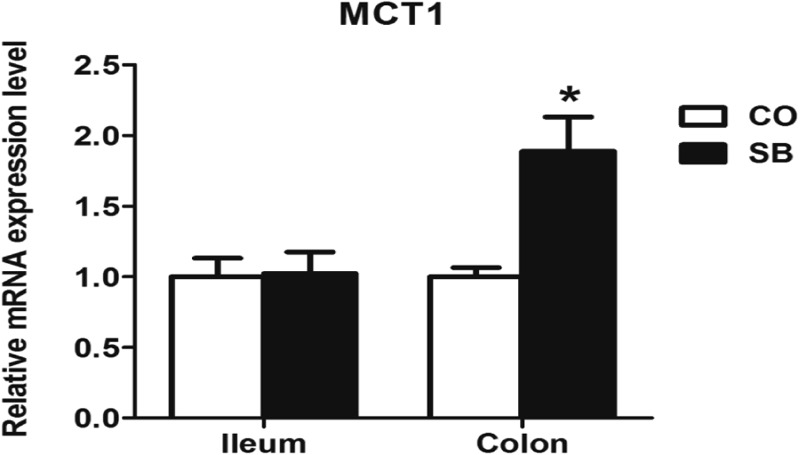
The relative gene expression of *MCT1* in the ileum and colon of pigs in the sodium butyrate (SB) and control (CO) groups. The values were calculated as the fold-expression change in SB group relative to CO group after normalization to the endogenous control, β-actin with formula 2^-ΔΔCt^. Values are expressed as mean ± SE, *n* = 6. ^∗^*P* < 0.05.

**FIGURE 4:**
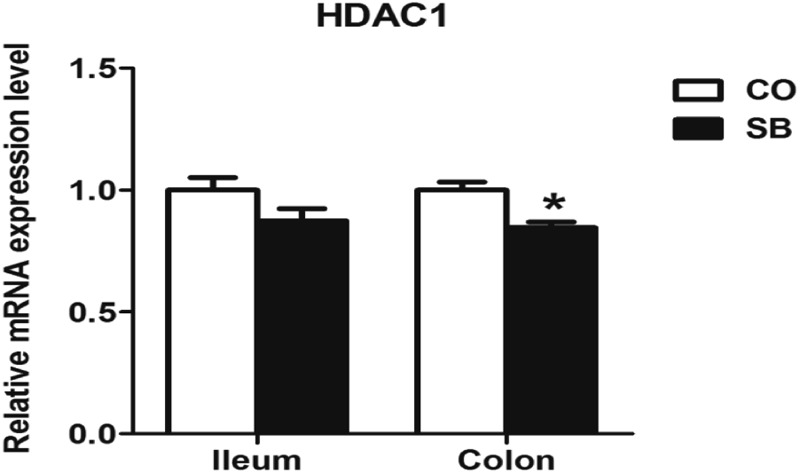
The relative gene expression of *HDAC1* in the ileum and colon of pigs in the sodium butyrate (SB) and control (CO) groups. The values were calculated as the fold-expression change in SB group relative to CO group after normalization to the endogenous control, β-actin with formula 2^-ΔΔCt^. Values are expressed as mean ± SE, *n* = 6. ^∗^*P* < 0.05.

Sodium butyrate treatment significantly up-regulated the gene expression of *MCT1* and down-regulated the expression of *HDAC1* in the colon (*P* < 0.05), while no changes of genes *MCT1* and *HDAC1* were found in the ileum between the two groups (**Figures 3, 4**).

### Gene Expression of Inflammatory Cytokines

**FIGURE 5:**
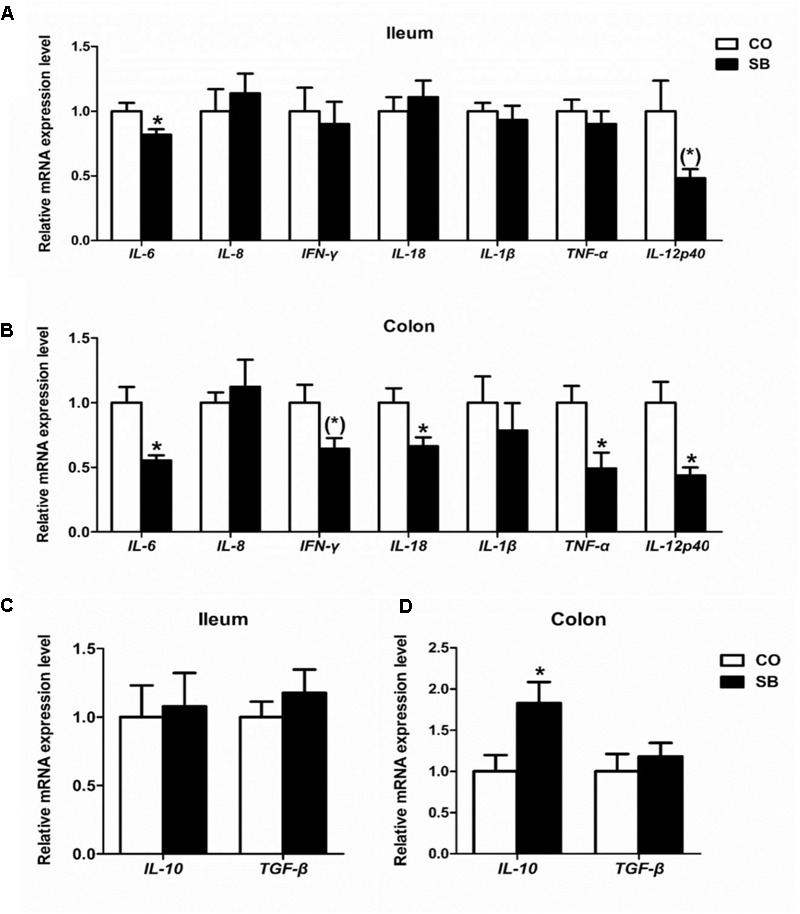
The relative gene expression of anti-inflammatory cytokines in the ileum **(A)** and colon **(B)**, and pro-inflammatory cytokines in the ileum **(C)** and colon **(D)** of pigs in the sodium butyrate (SB) and control (CO) groups. The values were calculated as the fold-expression change in SB group relative to CO group after normalization to the endogenous control, β-actin with formula 2^-ΔΔCt^. Values are expressed as mean ± SE, *n* = 6. ^∗^*P* < 0.05, (^∗^)0.05 ≤*P* < 0.1.

In the ileum, SB infusion significantly down-regulated the gene expression of pro-inflammatory *IL-6* (*P* < 0.05), and tended to decrease the gene expression of pro-inflammatory *IL-12p40* (*P* = 0.068), while had no effect on the gene expression of the other cytokines targeted in this study (**Figures 5A,C**). In the colon, SB treatment decreased the gene expression of pro-inflammatory *IL-6, IL-18, TNF-α*, and *IL-12p40* (*P* < 0.05), and tended to decrease the gene expression of pro-inflammatory *IFN-γ* (*P* = 0.059), while increased the gene expression of anti-inflammatory *IL-10* (*P* < 0.05). No difference in the gene expression of pro-inflammatory *IL-8* and *IL-1β* and anti-inflammatory *TGF-β* was observed between the two groups (**Figures 5B,D**).

### Expression of Tight Junction Protein- and Intestine Development-Related Genes

**FIGURE 6:**
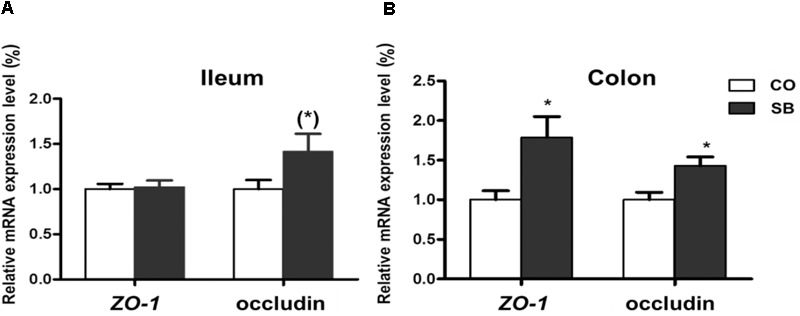
The relative gene expression of tight junction protein in the ileum **(A)** and colon **(B)** of pigs in the sodium butyrate (SB) and control (CO) groups. The values were calculated as the fold-expression change in SB group relative to CO group after normalization to the endogenous control, β-actin with formula 2^-ΔΔCt^. Values are expressed as mean ± SE, *n* = 6. ^∗^*P* < 0.05, (^∗^)0.05 ≤*P* < 0.1.

**FIGURE 7:**
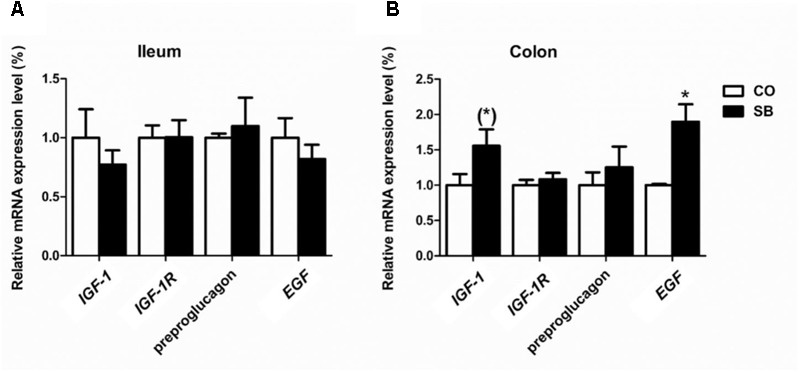
The relative gene expression of intestine development-relative gene in the ileum **(A)** and colon **(B)** of pigs in the sodium butyrate (SB) and control (CO) groups. The values were calculated as the fold-expression change in SB group relative to CO group after normalization to the endogenous control, β-actin with formula 2^-ΔΔCt^. Values are expressed as mean ± SE, *n* = 6. ^∗^*P* < 0.05, (^∗^)0.05 ≤*P* < 0.1.

In the ileum, SB infusion tended to increase the gene expression of occludin (*P* = 0.094), while had no effect on the expression of the other tight junction protein and intestine development targeted genes in this study (**Figures 6A, 7A**). In the colon, the gene expression of *ZO-1* and occludin in the SB group was higher than that in the CO group. SB infusion up-regulated the expression of intestine development-related gene *EGF* (*P* < 0.05) and tended to increase the gene expression of *IGF-1* (*P* = 0.081), whereas had no effects on the expression of genes *IGF-1R* and preproglucagon (*P* > 0.10) (**Figures 6B, 7B**).

### Correlation Between Colonic Microbial Composition and Inflammatory Cytokines-, Tight Junction Protein-, and Intestine Development-Related Gene Expression

**FIGURE 8:**
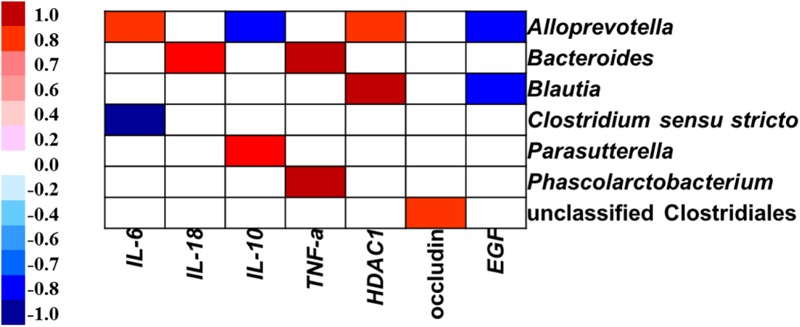
Correlation analysis between colonic microbial composition (relative abundance of genus higher than 0.1%) and inflammatory cytokines-, tight junction protein-, and intestine development-related gene expression which were significantly affected by sodium butyrate infusion in the colon of pigs, *n* = 6. The color represents a significant correlation (*P* < 0.05), and the intensity of the colors represents the degree of association. Red represents significant positive correlation, blue represents significantly negative correlation.

Pearson’s correlation analysis showed that the gene expression of *IL-6* was positively associated with the abundance of *Alloprevotella*, but negatively related to the proportion of *Clostridium sensu stricto* in the colon (**Figure 8**). The gene expression of *IL-18* was positively correlated with the abundance of *Bacteroides.* The *IL-10* expression level was positively associated with the abundance of *Parasutterella*, but negatively related to the relative abundance of *Alloprevotella.* The *TNF-α* expression level was positively related to the abundance of *Bacteroides* and *Phascolarctobacterium.* The gene expression of *HDAC1* was positively correlated with the abundance of genera *Alloprevotella* and *Blautia.* The gene expression of occludin was positively correlated with the abundance of unclassified Clostridiales. The *EGF* expression level was negatively correlated with the abundance of *Alloprevotella* and *Blautia* in the colon.

## Discussion

Butyrate, a SCFA, is mainly produced by microbial fermentation of diet fiber in the large intestine of monogastric animals. Butyrate is an important energy source for intestinal enterocytes and can be quickly absorbed by the epithelial cells. In the present study, we investigated the effects of intravenous infusion with SB on colonic microbiota, intestinal mucosal immunity, and intestinal development in normal growing pigs. We found that intravenous infusion with SB impacted the colonic bacterial community, and played beneficial roles in regulating the colonic mucosal immune response and the intestinal development.

Several studies showed that SB supplementation in the diet systematically resulted in a positive consequence of improved gut villous-crypt architecture on the host digestive efficiency and body weight in pig model (Galfi and Bokori, 1990; Wen et al., 2012; Huang et al., 2015). Moreover, this beneficial role in gut villous-crypt architecture was also observed in pig (Bartholome et al., 2004) and murine (Murakoshi et al., 2011) models with parenteral SB, however, the body weight was not affected. Similarly, the present study showed that no difference was found between the two groups in the pigs’ body weight, which might be due to the short-term treatment of SB.

Our previous study showed that oral intervention with SB increased the richness and diversity of colonic microbiota in newborn piglets (Xu et al., 2016). Huang et al. (2015) also found that dietary SB increased the microbial diversity and the abundance of Clostridiaceae, Ruminococcaceae, and Lachnospiraceae in the colon of weaned piglets. Similarly, in the present study, intravenous infusion with SB modified the colonic bacterial community significantly increased the bacterial richness. In addition, we also found that the abundance of unclassified Clostridiales, unclassified Ruminococcaceae, *Clostridium sensu stricto*, and *Anaerotruncus* that were belonging to Clostridiales order increased by SB infusion. The triggers for changes in bacterial community compositions are currently unknown, but parenteral SB may be indirectly involved via changes in the gut physiology of animals in response to SB infusion. In the present study, intravenous infusion with SB affected the gut barrier function through regulating the immune response, thus may indirectly modify the gut bacterial community. Similarly, this indirectly regulating role was also found in a previous study suggesting systemic LPS could alter ruminal environment and ruminal microbiota composition in dairy cattle (Jing et al., 2014). In the current study, parenteral SB treatment had no effects on the SCFA concentrations in the colon, which was in agreement with the fact that there was no difference in the number of total bacteria between the two groups. However, PCoA of colonic bacterial communities showed that the microbial clusters of the SB and CO groups were separated to some extent. The possible reason for the similar metabolic activity of the microbial community between the two groups may be that different microbial composition can lead to similar functions due to functional redundancy.

Sodium butyrate infusion deeply affected the colonic mucosal immune response with low impact on the ileum, which suggests that the butyrate in the circulation system may be more absorbed and utilized by the enterocytes in the colon than in the ileum. SCFAs can diffuse across the epithelial cell membrane, but SCFA absorption by the enterocytes is also mediated by the MCT1 (Halestrap and Meredith, 2004). Thus, *MCT1*, as the key transport element to transport butyrate in intestinal epithelial cells (Borthakur et al., 2008), was targeted to detect the mRNA expression. Indeed, the present study showed that SB infusion increased the gene expression of monocarboxylate transporters *MCT1* in the colon, whereas no change was found in the ileum. Emerging evidences suggest that gene expression of *MCT1* is increased by SCFA especially the butyrate both in the rumen of ruminants and the colon in the human (Borthakur et al., 2008; Naeem et al., 2012). Although SCFAs can diffuse across the epithelial cell membrane, these findings, to some extent, indicate that the absorption of butyrate in the circulation system may be increased by the enterocytes in the colon, and further impact the intestinal mucosal function.

*In vitro* cell culture experiments showed that butyrate plays a role in modulating immune systems via the inhibition of HDACs and down-regulation of the pro-inflammatory mediators such as *IL-6, IL-8, TNF-α*, and *IL-12p40* (Chang et al., 2014; Zhang et al., 2016). Infusion SCFAs to the distal ileum of growing pigs after oral antibiotics markly decreased the contents of IL-8 and TNF-α in the ileum and the relative mRNA abundance of pro-inflammatory cytokine *IL-8* in both ileum and colon (Diao et al., 2017). The present study showed that parenteral butyrate significantly down-regulated the gene expression of *HDAC1*, inhibited the gene expression of pro-inflammatory cytokines *IL-6, IL-18, TNF-α*, and *IL-12p40* in the colon, which is consistent with the previous studies through gut intervention with SB (Xiong et al., 2016). Differ to the study through oral intervention with SB, in our study, only *IL-6* gene expression was decreased by SB infusion in the ileum. The possible reason may be the limited butyrate absorption by ileal enterocytes from parenteral circulation system. In addition, the short-term treatment in this study might weaken its effect.

In this study, SB infusion significantly increased the sIgA concentration in the colon, and only a trend to increase was observed in ileum. It is known that sIgA plays an important role in protecting intestine against enteropathogens adherence and penetration in the intestinal mucosal both in human and animal models (McGhee et al., 1992; Phalipon et al., 2002). A previous study also reported that butyric acid–supplemented parenteral nutrition significantly increased intestinal IgA level with the decrease of IFN-γ level in mice (Murakoshi et al., 2011). The inhibition of HDAC reportedly restrained the production of IFN-γ (Sealy and Chalkley, 1978), which is consistent with our results showing a decrease of i gene expression and a trend in decrease of the gene expression of *IFN-γ* in the colon with SB infusion. In addition, as a Th2 IgA-stimulating cytokines, *IL-10* was also up-regulated in the colon by parenteral butyrate in the present study. IL-10 is secreted by regulatory T-lymphocytes, monocytes, and macrophages, and mainly inhibits the production of pro-inflammatory cytokines such as TNF-α and IFN-γ (Gracie et al., 1999). Nevertheless, detection of lymphocyte proliferation was not attempted in this study and the regulating role of parenteral SB on the function of lymphocyte requires further investigation. Although the mRNA expression of inflammatory cytokines in the gut was down-regulated by SB infusion, all piglets kept health during the whole experimental period. To fully understand the regulating mechanism of parenteral SB on gut mucosal immunity, *in vivo* pathogenic challenge model is needed in further studies.

It is reported that Clostridia, as the main butyrate producer, is beneficial to the immunological development and the maintenance of gut homeostasis (Umesaki et al., 1999; Lopetuso et al., 2013). Previous study reported that the effect of oral butyrate on HDACs and inflammatory factors ultimately renders the intestinal immune system hypersensitive to the beneficial butyrate-producing bacteria (Chang et al., 2014), which is in agreement with our finding in the variation of microbial composition in the colon of SB group. In addition, Pearson’s correlation analysis in this study also showed that the gene expression of *IL-6* was negatively related to the proportion of *Clostridium sensu stricto* in the colon. While the anti-inflammatory role of gut microbes such as *Faecalibacterium prausnitzii, Lactobacillus* spp. received more and more attention by researchers (Sokol et al., 2008; Bäuerl et al., 2013), our study found the correlations between some other genera and gene expression of inflammatory cytokines, however, considering the different roles in immunity activation of different species or strains in the same genus, further studies are needed to confirm the direct interaction between them at the species or strain level.

It is clear that the intestinal barrier has essential position in the innate immune system by preventing the harmful bacteria and noxious substances (Hasnain et al., 2011). The intestinal barrier consists of intact epithelial monolayer with the intercellular junctions that maintain structural integrity and normal functions of the intestinal epithelium (Matter et al., 2005). The tight junction, which is considered to be essential for the maintenance of normal intestinal structure, consists of transmembrane proteins (occludin and claudins) and ZO-1 proteins. In this study, the addition of SB in the jugular vein significantly up-regulated the gene expression of *ZO-1* and occludin in the colon, whereas only a trend for increase of gene expression of occludin was observed in the ileum, which might because the butyrate could be more absorbed by the enterocytes of the colon due to the increase of *MCT1* mRNA expression in the colon. These results were consistent with the previous findings showing that butyrate could enhance the barrier function of the colonic mucosa, maintain the integrity of intestine mucosa (Roediger, 1980; De Preter et al., 2011), and up-regulate the expression of tight junction proteins by enhancing the interaction between occludin-1 transcription factor SP1 and the promoter region (Wang et al., 2012). In addition, our correlation analysis showed that gene expression of occludin was positively correlated with the abundance of unclassified Clostridiales (including many butyrate producers), which is in agreement with the beneficial role of butyrate on the tight junction to some extent. The results above indicate that intravenous infusion with SB is beneficial for the intestinal integrity based on the detection of gene expression. However, further studies are needed to reveal the molecular mechanism of parenteral butyrate in promoting intestinal mucosa barrier on the protein level.

Researchers had also found that the growth factors such as GLP-2, IGF-1, and EGF are essential to the development of animal intestine. GLP-2 can not only promote the growth and re-repair of intestinal cells (Yazbeck et al., 2009), but also enhance intestinal absorption function via improving the intestinal morphology like stimulating crypt proliferation and villus growth (Drucker et al., 1996; Petersen et al., 2002). IGF-1 is another important regulator of the development of gut intestine and own similar effect on small intestine to GLP-2 and benefit to the small intestine integrity and absorption function (Houle et al., 1997; Alexander and Carey, 1999). IGF-1 is considered to play these functions mainly by binding with IGF-1R (Schober et al., 1990). EGF can promote DNA synthesis in intestinal mucosal cells and induce intestinal cell proliferation and differentiation (Xu et al., 1998). In the current study, a significant increase of *EGF* gene expression and a trend to increase of *IGF-1* gene expression were observed in the colon by the intravenous infusion with SB, while the gene expression levels of preproglucagon and *IGF-1R* in both segments were not affected. It was reported that parenteral SCFAs may influence small intestinal mucosal proliferation by stimulating secretion of proglucagon-derived peptides (GLP-2), and the relative gene expression of intestinal preproglucagon is strongly correlated with cellular proliferation during intestinal adaptation (Tappenden et al., 1997, 1998; Tappenden and Mcburney, 1998; Bartholome et al., 2004). It is not clear that why parenteral SB affect the *EGF* gene expression, however, a previous study showed that the EGF synthesis was remarkably increased in the proximal colon of the pigs fed with resistant starch due to the increase of microbial butyrate formation (Mentschel and Claus, 2003). Previous studies showed that the relative high abundance of bacteria such as *Bacillus* spp. in the intestine could up-regulated the gene expression of *GLP-2* and *IGF-1* in the small intestine of piglets (Xiang, 2011), however, in this study, the abundance of *Bacillus* was not affected by the SB infusion. In addition, our study found that the *EGF* expression level was negatively correlated with the abundance of *Alloprevotella* and *Blautia*. Nevertheless, further studies are needed to investigate the roles of these gut bacteria in regulating the host intestinal development.

In the present study, we chose a dosage of 2 mmol per day for SB infusion in a model of growing pigs (around 23.70 kg). In a model of neonatal piglets (around 1.77 kg) with 80% jejunoileal resection, 9 mmol/l butyrate infusion with total parenteral nutrition could improve the morphology and function of intestinal adaptation (Bartholome et al., 2004). Similarly, a previous study through oral supplement in a neonatal piglet model (Xu et al., 2016) found the dosage of 2 mmol SB per day was beneficial to the gut function and mucosal immunity. However, in our study, more significant effects on the colon were found as compared with the ileum, further study is needed to evaluate the optimal dosage of SB for enhancing the gut function and mucosal immunity at the whole intestinal level. In addition, long-term impact of parenteral SB requires further investigation.

## Conclusion

In conclusion, this study showed that intravenous infusion with SB increased the microbial richness and the relative abundance of genera related to Clostridiales order in the colonic digesta of pigs. Parenteral butyrate treatment up-regulated the mRNA expression of *IL-10, ZO-1*, Occludin, and *EGF*, and down-regulated the mRNA expression of *IL-6, IL-18, TNF-α, IL-12p40*, and *HDAC1* in colon. The results suggest that additional intravenous infusion with SB can modify colon microbial community, and to some extent, play a beneficial role in the gut health of pigs, which provides an alternative strategy for improving the intestinal health of pigs under the normal physiological condition.

## Author Contributions

YS and WZ conceived and designed the experiments. XC, JX, and YS performed the experiments and analyzed the data. XC and YS wrote the paper.

## Conflict of Interest Statement

The authors declare that the research was conducted in the absence of any commercial or financial relationships that could be construed as a potential conflict of interest.

## Footnotes

1 http://drive5.com/uparse/

2 http://rdp.cme.msu.edu/

3 http://www.mothur.org
