# Supplementary material for: Effects of Intravenous Infusion With Sodium Butyrate on Colonic Microbiota, Intestinal Development- and Mucosal Immune-Related Gene Expression in Normal Growing Pigs
Source: Front Microbiol. 2018 Jul 20;9:1652. doi: 10.3389/fmicb.2018.01652 (PMC6062594; doi:10.3389/fmicb.2018.01652)
Supplement: Supplementary file 1 [file Table_1.DOCX]

Supplementary Material

**Effects of intravenous infusion with sodium butyrate on colonic microbiota, intestinal development- and mucosal immune-related gene expression in normal growing pigs**

**Xue Chen, Jumei Xu, Yong Su*, Weiyun Zhu**

*** Correspondence:** Yong Su: yong.su@njau.edu.cn

**Supplementary Table 1.** Composition and nutrient analysis of experimental diet (as-fed basis).^1^

| Ingredients | Percentage (%) | Nutrient analysis |  |
| --- | --- | --- | --- |
| Corn | 65.00 | DE (MJ/kg) | 13.35 |
| Soybean meal | 22.00 | CP (%) | 16.80 |
| Wheat bran | 9.25 | NDF (%) | 11.88 |
| Soybean oil | 0.70 | ADF (%) | 4.12 |
| Lys | 0.18 |  |  |
| Thr | 0.01 |  |  |
| CaHPO_3_ | 0.69 |  |  |
| Rock powder | 0.87 |  |  |
| Salt | 0.30 |  |  |
| 1% Premix^2^ | 1.00 |  |  |

^1^DE, digestible energy; CP, crude protein; NDF, neutral detergent fiber; ADF, acid detergent fiber.

^2^Premix provided these amounts of vitamins and minerals per kilogram on an as-fed basis: vitamin A, 10,800 IU; vitamin D3, 4,000 IU; vitamin E, 40 IU; vitamin K3, 4 mg; vitamin B1, 6 mg; vitamin B2, 12 mg; vitamin B6, 6 mg; vitamin B12, 0.05 mg; biotin, 0.2 mg; folic acid, 2 mg; niacin, 50 mg; D-calcium pantothenate, 25 mg; Fe, 100 mg as ferrous sulfate; Cu, 150 mg as copper sulfate; Mn, 40 mg as manganese oxide; Zn, 100 mg as zinc oxide; I, 0.5 mg as potassium iodide; and Se, 0.3 mg as sodium selenite.
