# Supplementary material for: Effects of Intravenous Infusion With Sodium Butyrate on Colonic Microbiota, Intestinal Development- and Mucosal Immune-Related Gene Expression in Normal Growing Pigs
Source: Front Microbiol. 2018 Jul 20;9:1652. doi: 10.3389/fmicb.2018.01652 (PMC6062594; doi:10.3389/fmicb.2018.01652)
Supplement: Supplementary file 2 [file Table_2.DOCX]

Supplementary Material

**Effects of intravenous infusion with sodium butyrate on colonic microbiota, intestinal development- and mucosal immune-related gene expression in normal growing pigs**

**Xue Chen, Jumei Xu, Yong Su*, Weiyun Zhu**

*** Correspondence:** Yong Su: yong.su@njau.edu.cn

**Supplementary Table 2.** List of the primers used in the present study.

| Genes | Primer sequences (5`→3') | Accession no. | Amplicon size (bp) |
| --- | --- | --- | --- |
| *MCT1* | F: CATCAACTACCGACTTCTG | XM 021088451.1 | 80 |
|  | R: TACTGGTCTCCTCCTCTT |  |  |
| *TNF-α* | F: CCACGCTCTTCTGCCTACTGC | NM 214022.1 | 168 |
|  | R: GCTGTCCCTCGGCTTTGAC |  |  |
| *IL-1β* | F: AGTGGAGAAGCCGATGAAGA | XM 021085847.1 | 113 |
|  | R: CATTGCACGTTTCAAGGATG |  |  |
| *IL-6* | F: CCTCTCCGGACAAAACTGAA | NM 001252429.1 | 118 |
|  | R: TCTGCCAGTACCTCCTTGCT |  |  |
| *IL-8* | F: TAGGACCAGAGCCAGGAAGA | NM 213867.1 | 92 |
|  | R: AGCAGGAAAACTGCCAAGAA |  |  |
| *IFN-γ* | F: TCCAGCGCAAAGCCATCAGTG | NM 213948.1 | 111 |
|  | R: ATGCTCTCTGGCCTTGGAACATAGT |  |  |
| *IL-18* | F: TATGCCTGATTCTGACTGTT | XM 005667327.2 | 260 |
|  | R: ATGAAGACTCAAACTGTATCT |  |  |
| *IL-12p40* | F: GATGCTGGCCAGTACACC | NM 214013.1 | 377 |
|  | R: TCCAGCACGACCTCAATG |  |  |
| *IL-10* | F: CTGCCTCCCACTTTCTCTTG | NM 214041.1 | 95 |
|  | R: TCAAAGGGGCTCCCTAGTTT |  |  |
| *TGF-β* | F: GAAGATGCTTGGAGCTGAGG | XM 013978322.2 | 121 |
|  | R: TGGGACTTTGTCTTGGGAAC |  |  |
| *HDAC1* | F:CCAAGTACCACAGTGATGACTACATT | XM 013999116.2 | 135 |
|  | R:AGAACTCAAACAGGCCATCAAA |  |  |
| *ZO-1* | F: GAGGATGGTCACACCGTGGT | XM 021098896.1 | 169 |
|  | R: GGAGGATGCTGTTGTCTCGG |  |  |
| occludin | F: ATGCTTTCTCAGCCAGCGTA | XM 005672525.3 | 176 |
|  | R: AAGGTTCCATAGCCTCGGTC |  |  |
| preproglucagon | F: ACTCACAGGGCACGTTTACCA | XM 005671883.3 | 150 |
|  | R: AGGTCCCTTCAGCATGTCTCT |  |  |
| *EGF* | F: ATCTCAGGAATGGGAGTCAACC | XM 021100462.1 | 166 |
|  | R: TCACTGGAGGATGGAATACAGC |  |  |
| *IGF-1* | F: CTGAGGAGGCTGGAGATGTACT | XM 005664199.3 | 137 |
|  | R: CCTGAACTCCCTCTACTTGTGTTC |  |  |
| *IGF-1R* | F: ATGGAGGAAGTGACAGGGACTA | XM 021082920.1 | 116 |
|  | R: GTGGTGGTGGAGGTGAAGTG |  |  |
| β-actin | F: AGAGCGCAAGTACTCCGTGT | XM 021086047.1 | 68 |
|  | R: ACATCTGCTGGAAGGTGGAC |  |  |
| *GAPDH* | F: ATCCTGGGCTACACTGAGGA | NM 001206359.1 | 130 |
|  | R: TGTCGTACCAGGAAATGAGCT |  |  |
